# Chronic low back pain: a prospective study with 4 to 15 years follow-up after a multidisciplinary biopsychosocial rehabilitation program

**DOI:** 10.1186/s12891-022-05963-w

**Published:** 2022-11-11

**Authors:** Franziska R. Ochsenkuehn, Alexander Crispin, Martin B. Weigl

**Affiliations:** 1 Department of Orthopaedics and Trauma Surgery, Musculoskeletal University Center Munich (MUM), University Hospital, LMU Munich, Munich, Germany; 2 Klinik Für Anästhesiologie, Caritas-Krankenhaus St. Josef, Regensburg, Germany; 3 Institute for Medical Information Processing, Biometry, and Epidemiology, LMU Munich, Munich, Germany

**Keywords:** Chronic low back pain, Multidisciplinary biopsychosocial rehabilitation, Exercise therapy, Cognitive behavioral therapy, Observational study, Cohort study, Long-term effects, Outcome assessment

## Abstract

**Background:**

Multidisciplinary biopsychosocial rehabilitation (MBR) in patients with chronic low back pain (CLBP) is superior to less intensive treatments for at least one year, but the long-term course of the disease is largely unknown. The primary aim of this study was to describe the long-term course of an MBR in relation to pain, disability, and quality of life from the beginning of an MBR to between 4 to 15 years after participation. The secondary aim was to explore the long-term course of an MBR in relation to physiological outcomes of functioning.

**Methods:**

This was a observational study conducted at a university hospital. The cohort consisted of participants of a 3-week, CLBP-specific MBR program between August 2001 and January 2013. The North American Spine Society questionnaire (NASS) pain and disability scale was the primary patient -reported outcome measure (PROM). The NASS neurogenic symptoms scale and the Short-Form 36 (SF-36) health survey were secondary PROMs. Patients were assessed before entry to the MBR (T0), at entry (T1), at discharge (T2) and 4 to 15 years after discharge (T3). Effects were quantified by effect size (ES). Score differences were tested for significance using parametric or non-parametric tests and linear mixed models.

**Results:**

Of 299 consecutive patients from the MBR program, 229 could be contacted. Of these, 84 declined participation, five did not meet the inclusion criteria, and 26 had incomplete data. Thus, 114 patients were included. The mean follow-up time was 9.2 years. At T3, patients exhibited beneficial effects for NASS pain and disability with a moderate ES (ES = 0.63; *p* < 0.001). The NASS neurogenic symptoms scale was stable. The SF-36 scales showed an improvement in the bodily pain domain (ES = 1.02; *p* < 0.001), but no significant changes for physical functioning, physical role, general health, vitality, social functioning, emotional role, or mental health. The physical health component summary was improved (ES = 0.40, *p* = 0.002), and the mental health summary was unchanged. The linear mixed model analysis confirmed improvements in pain and disability between T1 and T3 (*p* = 0.010).

**Conclusions:**

The results of this study suggest that there is a long-term benefit of MBR participation in patients with CLBP.

## Introduction

Low back pain (LBP) is a common health condition with more than 500 million people globally affected at any one time [1]. It ranks first globally in years lived with disability statistics [1]. Following an acute episode of LBP, most patients recover within 12 weeks [2]. However, 10–20% of patients experience chronic and persistent LBP for more than 12 weeks [3]. Predictors for chronic low back pain (CLBP) include symptom-related factors (previous episodes and back pain intensity), life-style factors (low levels of physical activity), psychological factors (depressive symptoms and fear-avoidance beliefs) and social factors (low educational level and work dissatisfaction) [4].

Multidisciplinary biopsychosocial rehabilitation (MBR) addresses the multiple factors that contribute to CLBP [5]. They combine active physical therapy, psychological interventions, and patient education [5]. A systematic review of randomized, controlled, clinical trials has confirmed the superiority of MBR programs compared with physical therapy treatment or typical care [5]. Follow-ups in clinical trials have shown that MBR improves pain and physical functioning for at least one year [5]. Clinical practice guidelines recommend MBR for CLBP if less intensive treatments have not been successful [6–8].

However, there is little evidence for the long-term effects of MBR (more than one year following treatment) [9–13]. Some of the studies with long-term follow-ups are outdated [10, 11], and have very low sample sizes [12], while others focused on a young patient population [10, 12] or have very low follow-up rates [13].

Knowledge concerning the long-term course after an MBR would help clinicians counsel candidates for an MBR about the course patients could expect when they become older. If the long-term course in pain and disability is favorable, this could assure patients who are worried that CLPB necessarily gets worse with age and help motivate them to participate in an MBR. For health insurances, knowledge concerning long-term course outcomes could support them in deciding whether to provide coverage for MBR programs.

Previous long-term studies have measured outcomes in functioning via patient self-reporting, but have not added physiological measures [9–13]. If physiological parameters can be improved by an MBR, then this may contribute to an improvement in pain and disability. Moreover, this knowledge could help to adapt and improve the contents of an MBR.

Therefore, we designed a long-term follow-up study of patients with CLBP who had participated in an MBR. A previous evaluation of this particular MBR demonstrated that patients improved for at least 12 months after treatment [14]. In this study, the objective was to describe the long-term course of pain, disability, and quality of life in patients with CLBP after participation in an MBR. We hypothesized that the patients would have less pain and disability and a stable quality of life at the long-term follow-up compared to before their MBR.

## Methods

### Aim

The primary aim of this study was to describe the long-term course of an MBR in relation to pain, disability, and quality of life from the beginning of an MBR to between 4 to 15 years after participation. The secondary aim was to explore the long-term course of an MBR in relation to physiological outcomes of functioning.

### Study design

In this observational study, the data were prospectively collected from a cohort of patients that participated in an MBR. The schedule and measures for the assessments before the 3-week MBR (T0), at the beginning of the MBR (T1) and at the end of the MBR (T2) were defined before data collection started. The mean waiting time between T0 and T1 was 139 (± 159) days. The long-term follow-up occurring between 4 to 15 years after the MBR (T3) was defined only after the data at T0, T1, and T2 had already been collected. The study was approved by the institutional review board at the medical faculty of the Ludwig Maximilian University Munich (project number 632–16). The study was conducted in accordance with the Declaration of Helsinki. All patients signed informed consent forms. The study was not preregistered.

### Setting

University Hospital, Ludwig Maximilian University Munich, day clinic of the Department of Orthopedics, Physical Medicine and Rehabilitation.

### Participants

Patients who had participated at least 4 years previously were selected from a database of all MBR patients. A member of the study team attempted to call all selected patients. He explained the content of the study and evaluated patients for inclusion in the study. Patients with severe illness at T3 that could have caused disability that would have masked the long-term course of pain and disability were excluded. Further exclusion criteria were insufficient German language skills to fill in questionnaires and participation in another MBR in the 12 months before T3. Furthermore, patients were excluded if the primary outcome measure was missing at T1, T2 or T3. If a patient was interested in participating, information and informed consent were sent to the patient by mail.

The included patients were retired or older working adults as this program complemented a pre-existing MBR program at the University Hospital Munich for young, working adults [15].

A subgroup of 37 patients was invited for reassessment using the same physiological measures that had been conducted at T1 and T2. To form a representative subgroup, the whole cohort was divided into eight groups according to gender (male/female), age (above/below the median), and participation in a one-week MBR refresher approximately one year after the MBR (yes/no). Each group had between 6 and 27 patients. One third of the patients from each subgroup was randomly selected.

### Intervention

The clinic has been offering this LBP-specific MBR program since 2001. The recommendation for participation in the MBR was based on an interdisciplinary assessment. The criteria for participation in the MBR have been presented elsewhere [14].

The intervention was a 3-week MBR with three treatment days every week and a total of 44 treatment hours [14].

**Fig. 1:**
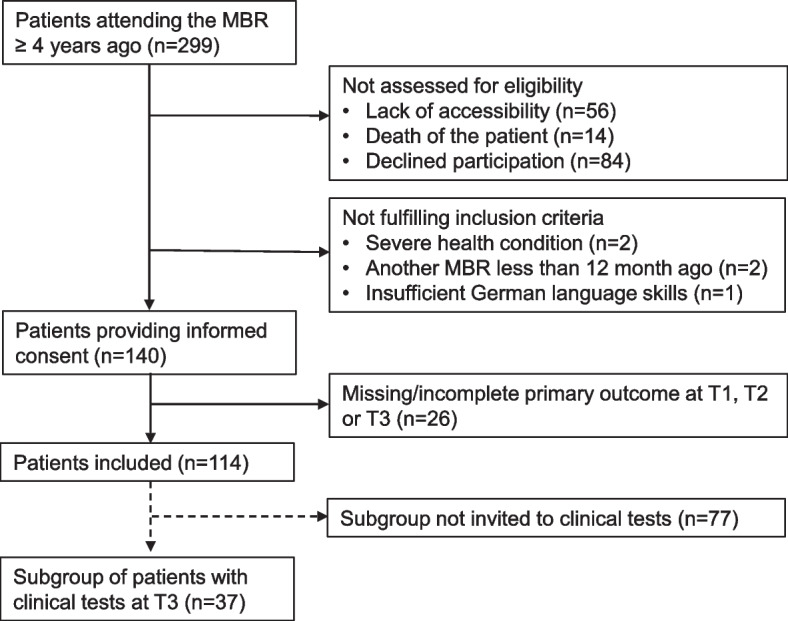
Patient flow diagram. MBR: multidisciplinary biopsychosocial rehabilitation. T1: Start of the MBR; T2: End of the MBR; T3: Long-term follow-up occurring 4 to 15 years after the MBR.

The rehabilitation team included a specialist in Physical and Rehabilitation Medicine, a psychologist, a physiotherapist, and an occupational therapist. Figure 1 provides an overview of the treatments. The MBR included several measures for maintaining treatment effects over the long-term. All health professionals provided written informational materials to the patients. In weekly interdisciplinary group meetings, patients were asked by the rehabilitation team to define their personal goals for the next week. All health professionals encouraged patients, during personal conversations, to continue the most helpful contents of the MBR after the program was completed. At the end of the MBR, the patients, together with the health professionals, developed patient-specific, long-term goals by applying the SMART-technique (specific, measureable, achievable, relevant, time bound) [16]. More details of this intervention have been described previously [14].

### Data collection

Patients completed questionnaires at T0, T1, and T2 on-site in the clinic on the same day as the examination for the physiological measures. At T3, the questionnaires were sent by postal mail. The study team provided pre-addressed and stamped envelopes and asked the patients to return the questionnaires by postal mail. If patients did not return the questionnaire after two weeks, the research assistant called the patient by phone and asked for the questionnaires to be returned.

Data were manually transferred from the questionnaires into a database by a member of the study team. Unclear responses, e.g., those with 2 or more crosses, were considered missing values. For data cleaning, two members of the study team independently controlled all data visually for duplicate data, duplicate rows, missing values, or obvious typing errors. Then, statistical tests were calculated to ensure that all data were within the specified range. In the next step, the lower and upper extreme scores of all scales and physiological measures were identified. The data from these patients were validated against the original data in the questionnaires and the forms for the physiological measures.

### Measures of participant characteristics

Anxiety and depression were measured using the Hospital Anxiety and Depression Scale (HADS) [17, 18] at T1 and T3. This instrument has two scales with 7 items each that range from 0 (best health) to 21 (worst health). According to validation studies, patients scoring less than 8 represent no cases of depression/anxiety, while scores from 8 to 10 are doubtful cases of depression/anxiety, and scores greater than 10 are cases of depression/anxiety [17, 18].

The number of comorbidities was counted via the validated Self-administered Comorbidity Questionnaire [19] at T1 and T3.

Patient education was assessed at T1 by a single written question.

## Outcome measures

### Patient reported outcome measures (PROMs)

The primary outcome was pain and disability as measured by the North American spine society lumbar spine outcome assessment Instrument (NASS) pain and disability scale [20, 21] at T3. The NASS scale included 11 items. Questions were scored from 1 (best health) to 6 (worst health). The scale score was determined by calculating the arithmetic mean of the items. Several secondary outcomes were also measured. Neurogenic symptoms were evaluated by the corresponding NASS scale with six items [20, 21]. Generic, health-related quality of life was measured by the Short-Form (SF-36) [22, 23]. This is comprised of 36 questions that addressed the following eight dimensions: physical functioning, physical role, bodily pain, general health, vitality, social functioning, emotional role, and mental health. Each scale ranged from 0 (worst health) to 100 (best health). The first four subscales constitute the Physical Component Summary and the latter four scales the Mental Component Summary.

### Physiological Measures

Physiological measures were applied according to standardized written protocols. A highly experienced physiotherapist (with more than 20 years of professional experience) trained the examiners. At T1 and T2, physiotherapists or occupational therapists conducted the tests. At T3, the examiner was a doctoral medical student.

Isometric muscle strength during flexion and extension of the knee joints was measured with a hand-held pull gauge (Wagner Instruments, Greenwich, USA) connected to a belt [24]. The patient sat in a standardized upright position with 90° knee flexion. The tester placed the belt of the pull-gauge close to the ankle joint. Next, the tester held the pull-gauge in a stable position and asked the patient to press the leg slowly forward against the band with increasing force to measure knee extension strength, and to press backwards for knee flexion strength. The unit of measure was the kilopond and the reliability was high [24].

The timed-up-and-go-test assesses mobility, as well as static and dynamic balance, and is associated with the risk of falls. The patient sits on a chair, stands up, walks three meters, turns around, walks back to the chair and sits down again. The unit of measure is seconds. It has high reliability and is frequently applied in older persons [25]. It is the most frequently used functional physiological outcome measure for LBP patients with degenerative disease in clinical studies [26]. Degenerative changes are a potential contributing factor to low back pain and disability in our population of older LBP patients.

The 6-min-walk-test assesses a patient's physical aerobic endurance and walking ability. Before and during the test the patient received standardized instructions. The unit of measure was the walking distance in meters [27]. The 6-min-walk-test has been used in various populations including patients with CLBP [28].

## Statistical methods

### Primary analysis

The course of pain and function was described by the scores from the PROMs and physiological measures. Changes from T1 to the follow-ups at T2 and T3 were quantified by effect size (ES) [29]. The ES was calculated with the formula: ES = (Mean (T1) – Mean (follow-up))/SD (T1). An ES of 0.2 was considered a small effect, 0.5 a moderate effect, and 0.8 a large effect [29]. A clinically meaningful effect was assumed for an ES of more than 0.3, provided that no instrument-specific study was available that evaluated an instrument-specific ES [30].

Significant changes in scores were tested by means of paired t-tests if data were normally distributed. Otherwise, the Wilcoxon signed-rank test was applied. The significance level was set at *p* < 0.05. The significance tests were confirmatory for changes in the primary outcome NASS pain + disability between T1 and T3. All other significance tests were exploratory.

No data was imputed. Only cases with valid scores for the variable under consideration at T1, T2, and T3 were included. In the analysis of the SF-36 scales, only cases with valid scores in all scales were included.

### Other analysis

Results of the primary outcome measure NASS pain + disability and the SF-36 physical and mental health component summaries were corroborated using linear mixed models that allowed for adjustment of the possible confounders of gender and age. These models used a random intercept per patient and the fixed effects of time (T0, T1, T2, and T3), age, gender, and baseline NASS or SF-36 scores. The formula used was:

y_i(t) = β_0i + β_1 × (t = T0) + β_2 × (t = T2) + β_3 × (t = T3) + β_4 × y_i (t = T1) + β_5 × age + β_5 × male.

Where:

y_i (t): individual value of the outcome variable of patient i at time t.

β_0i: random intercept for patient i.

β_1,β_2,…,β_5: regression coefficients for the fixed effects (time, baseline score, age, male sex).

A longer follow-up could result in a selection bias for healthier patients as the longer the observation period, the more likely it is that no current contact information would be available because the patient would have moved to a retirement home or died. This risk of selection bias was addressed via a subgroup analysis of patients with differing lengths of follow-up time. Patients were divided into groups with more or less than 9 years of follow-up time, as 9 years was the nearest year to the median.

Statistical analyses were performed with the software package SPSS 25.0 (IBM Corp., Armonk, NY, USA) for Windows. ES values were calculated using Microsoft EXCEL 2016. These analyses were performed using SAS 9.4 for Windows (SAS Institute, Cary, NC, USA).

### Drop-out analysis

We compared the age and gender of patients who were included in the long-term follow-up analyses to patients who were not included in the follow-up.

### Sample size considerations

In an earlier study, the MBR had an average ES of 0.36 in the primary outcome pain + disability after one year [14]. We assumed a small decrease in the ES from 0.36 to 0.30 over the course of the long-term follow-up. An ES ≥ 0.30 became statistically significant at a sample size of n ≥ 70 [30]. When the study began, 299 candidates for participation were in the database. Due to our experience in previous observational cohort studies, we were confident in our ability to include more than the minimum of 70 patients in the primary analyses.

## Results

### Patients

In December 2016, of the 299 patients that had been participating in the MBR at least four years before, there was no current contact information available for 56 patients, and we received information about the patient's death for 14 patients. The remaining 229 patients were contacted by phone between December 2016 and July 2017. Of these, 84 declined participation. Two were excluded due to severe illness, two due to participation in another MBR less than 12 months previously, and one due to insufficient German language skills. Of the remaining 140 patients, 26 were excluded due to incomplete data in the primary outcome measure. Thus, 114 patients (38.1%) were included in the primary analysis.

**Fig. 2:**
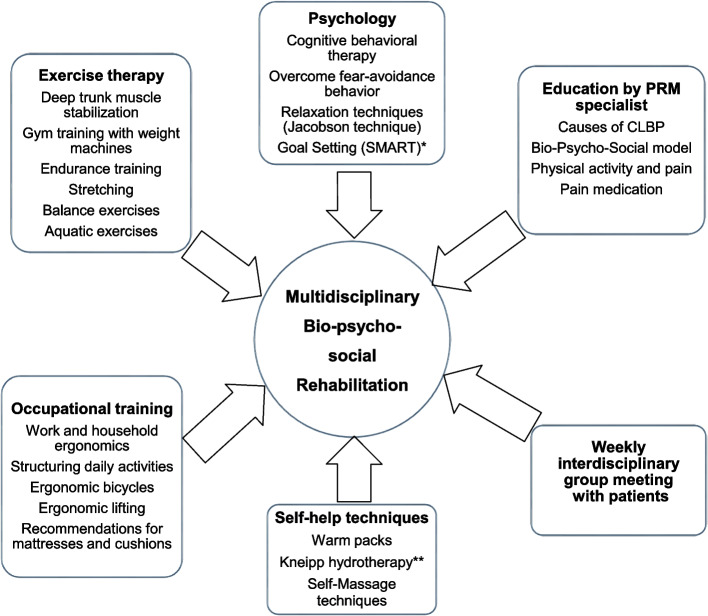
Multidisciplinary biopsychosocial rehabilitation program. * SMART goals: specific, measureable, achievable, relevant, time bound [18]. **Kneipp hydrotherapy: repeated cold water stimulations.

A subgroup of 37 patients was also invited for reassessment of physiological measures. A study inclusion flow diagram is presented in Fig. 2.

The participant characteristics of the 114 patients included in the study are presented in Table 1.

**Table 1:** Participant characteristics at T1^1^ and T3^2^ (*n* = 114)

Characteristic	Value Entry (T1)	Value Follow-up (T3)
Female, *n* (%)	78 (68.4)	Unchanged
Age (years), mean (SD^3^)	62.8 (8.3)	72.0 (7.7)
Follow-up (years), mean (SD)	n.a	9.2 (3.5)
**Education, n (valid %)**		
Basic school (9 years)	38 (33.3)	Unchanged
Middle school (10 years)	44 (38.9)	Unchanged
High school (> = 12 years)	12 (10.6)	Unchanged
University/ Technical college	18 (15.9)	Unchanged
**Co-morbidities, n (valid %)**		
None	24 (21.1)	20 (17.5)
1	43 (37.7)	30 (26.3)
2	27 (23.7)	24 (21.1)
3	11 (9.6)	23 (20.2)
≥ 4	2 (1.8)	17 (14.9)
Spine surgery after T1, *n* (valid %)	n.a	11 (9.6)
**Depression (HADS**^**4**^**)**		
Mean (SD)	5.15 (3.1)	5.49 (4.0)
doubtful/case of depression, *n (valid %)*	11/7 (10.7/6.8)	20/11 (19.4/10.7)
**Anxiety (HADS**^**4**^**)**		
Mean (SD)	6.24 (3.48)	6.45 (3.60)
doubtful/case of anxiety, *n (valid %)*	24/12 (23.3/11.7)	23/14 (22.3/13.6)

^1^T1: Start of the Multidisciplinary Biopsychosocial Rehabilitation program

^2^T3: Long-term follow-up (4.2 – 15.7 years after T1)

^3^SD: Standard deviation

^4^HADS: Hospital Anxiety and Depression Scale; Patients scoring less than 8 represent no cases of depression/anxiety, 8 to 10 are doubtful cases of depression/anxiety, and greater than 10 are cases of depression/anxiety [18]

The mean patient age was 62.8 (± 8.3) at T1 and 72.0 (± 7.7) years at T3. Females accounted for 68.4% of participants. Patients were followed for a mean time of 9.2 (± 3.5) years.

### Outcome of proms at T1, T2 and T3

Table 2 presents the course of the PROMs from T1 to T3. When testing for normality paired t–tests were used for all scales except the SF-36 physical and emotional role scales. For these, the Wilcoxon signed rank-test was used.

**Table 2:** Patient-reported outcome measures from entry to treatment to long-term follow-up after 4–15 years (*n* = 114)

	**Entry (T1**^**1**^**)**		**Discharge (T2**^**2**^**)**				**Follow-Up (T3**^**3**^**)**			
	**Mean**	**(SD**^**4**^**)**	**Mean**	**(SD)**	**Effect size**	***p*-value**	**Mean**	**(SD)**	**Effect size**	***p*-value**
**Primary outcome**										
NASS^5^, pain + disability	3.05	0.72	2.59	0.75	0.64	< 0.001	2.60	0.84	0.63	< 0.001
**Secondary outcomes**										
NASS, Neurogenic symptoms (n = 112)	2.41	1.10	1.96	0.96	0.41	< 0.001	2.33	1.13	0.07	0.51
**Short-Form 36 (n = 99)**										
Physical functioning	63.02	16.82	69.02	18.33	0.36	< 0.001	63.05	21.77	0.00	0.988
Physical role	40.57	38.66	51.35	39.14	0.28	0.012	50.00	42.86	0.24	0.067
Bodily pain	38.85	14.60	48.64	16.96	0.67	< 0.001	53.70	20.44	1.02	< 0.001
General health	53.17	16.92	56.44	16.05	0.19	0.022	52.88	18.68	-0.02	0.863
Vitality	49.39	15.32	56.80	16.66	0.48	< 0.001	50.94	17.81	0.10	0.346
Social functioning	72.98	21.99	79.17	20.75	0.28	0.001	74.12	22.11	0.05	0.640
Emotional role	78.79	34.82	86.53	29.32	0.22	0.051	74.07	39.71	-0.14	0.272
Mental health	68.66	17.09	72.96	15.58	0.25	0.002	69.92	16.21	0.07	0.452
Physical component summary	35.28	7.67	38.34	8.61	0.40	< 0.001	38.37	10.02	0.40	0.002
Mental component summary	50.72	9.76	53.05	8.66	0.24	0.008	49.79	10.56	-0.10	0.381

^1^T1: Start of the multidisciplinary biopsychosocial rehabilitation

^2^T2: End of the multidisciplinary biopsychosocial rehabilitation

^3^T3: Long-term follow-up occurring 4–15 years after the multidisciplinary biopsychosocial rehabilitation

^4^SD: Standard deviation

^5^NASS: North American Spine Society Outcome Instrument

At T3, NASS pain + disability showed a moderately beneficial effect (ES = 0.63, *p* < 0.001). Further significant improvements were found for the SF-36 bodily pain scale and the SF-36 physical health component summary. The other scales showed no significant differences from T1.

### Physiological outcomes at T1, T2 and T3

Physiological outcomes were measured in a subgroup of 37 patients. The results are presented in Table 3.

**Table 3:** Physiological outcomes from entry to treatment to long-term follow-up after 4–15 years (*n* = 37)

	**Entry (T1**^**1**^**)**		**Discharge (T2**^**2**^**)**				**Follow-Up (T3**^**3**^**)**			
	**Mean**	**(SD**^**4**^**)**	**Mean**	**(SD)**	**Effect** **size**	***p*-value**	**Mean**	**(SD)**	**Effect** **size**	***p*-value**
6-min walk, m	526.9	106.9	570.1	87.3	0.40	0.003	522.0	89.9	-0.05	0.305
Timed-up-And-Go, s	7.8	1.5	7.1	1.4	0.47	0.001	7.9	1.8	-0.07	0.634
**Isometric muscle strength, kp**^**5**^										
Knee extension, right	22.7	8.5	25.1	7.8	0.28	0.024	24.1	8.9	0.16	0.278
Knee extension, left	21.4	7.3	23.9	7.6	0.34	0.013	26.1	9.3	0.64	0.002
Knee flexion, right	15.0	5.9	17.3	5.8	0.39	0.001	16.9	6.1	0.32	0.027
Knee flexion, left	14.4	5.1	16.2	5.6	0.35	0.049	17.0	6.7	0.51	0.061

^1^T1: Start of the multidisciplinary biopsychosocial rehabilitation

^2^T2: End of the multidisciplinary biopsychosocial rehabilitation

^3^T3: Long-term follow-up occurring 4–15 years after the multidisciplinary biopsychosocial rehabilitation

^4^SD: Standard deviation

^5^kp: kiloponds

At T3, a beneficial effect for left knee extension (ES = 0.64; p = 0.002) and right knee flexion (ES = 0.32; *p* = 0.027) remained. Other physiological measures showed no significant differences between T1 and T3.

### Linear mixed models

The results of the linear mixed model analysis for the primary outcome parameter pain + disability is shown in Table 4.

**Table 4:** Mixed linear model for the North American Spine Society (NASS) pain + disability scale

Fixed effect	Visit	Estimate	*p*-value
Intercept		0.90	0.02
Time point	Before entry (T0)^2^	0.17	0.17
Time point	Entry (T1)^3^	0.00	
Time point	Discharge (T2)^4^	-0.72	< 0.0001
Time point	Follow-up (T3)^5^	-0.33	0.01
Baseline Score		0.65	< 0.0001
Age		0.01	0.07
Male		-0.13	0.18

^1^df: Degrees of freedom

^2^T0: Assessment before multidisciplinary biopsychosocial rehabilitation

^3^T1: Start of the multidisciplinary biopsychosocial rehabilitation

^4^T2: End of the multidisciplinary biopsychosocial rehabilitation

^5^T3: Long-term follow-up occurring 4–15 years after the multidisciplinary biopsychosocial rehabilitation

The pain + disability scale improved between T1 and T2 (*p* < 0.0001). The significance of the improvement persisted until T3 (*p* = 0.010). There was no change during the waiting time between T0 and T1. These results suggest that the improvements were due to the MBR and not to natural variations over the course of LBP.

The results for the SF-36 physical and mental component summaries are shown in Table 5.

**Table 5:** Mixed linear model for the Short-Form 36 component summaries

Fixed effect	Visit	Estimate	*p*-value
**Physical component summary**			
Intercept		53.75	0.00
Time point	Before entry (T0)^2^	0.28	0.75
Time point	Entry (T1)^3^	0.00	
Time point	Discharge (T2)^4^	2.63	< 0.0026
Time point	Follow-up (T3)^5^	1.25	0.20
Baseline Score		-2.18	< 0.0001
Age		-0.14	0.07
Male		0.57	0.67
**Mental component summary**			
Intercept		47.64	< 0.0001
Time point	Before entry (T0)^2^	-1.51	0.13
Time point	Entry (T1)^3^	0.00	
Time point	Discharge (T2)^4^	2.71	0.01
Time point	Follow-up (T3)^5^	-0.83	0.46
Baseline Score		-0.27	0.61
Age		0.06	0.54
Male		1.26	0.45

^1^df: Degrees of freedom

^2^T0: Assessment before multidisciplinary biopsychosocial rehabilitation

^3^T1: Start of the multidisciplinary biopsychosocial rehabilitation

^4^T2: End of the multidisciplinary biopsychosocial rehabilitation

^5^T3: Long-term follow-up occurring 4–15 years after the multidisciplinary biopsychosocial rehabilitation

Changes between T1 and T 3 were not significant (physical component: *p*-value = 0.20; mental component: *p*-value = 0.46).

### Subgroup analysis

In both subgroups of patients with follow-up periods of 4 to < 9 years and > 9 years, the NASS pain + disability scale showed significant improvements, with an ES = 0.46 for the shorter follow-up and an ES = 0.79 for the longer follow-up period (Table 6).

**Table 6:** Comparison of outcomes between patients with a follow-up of < 9 years and ≥ 9 years

			**Entry (T1**^**1**^**)**		**Follow-up (T3**^**2**^**)**			
	**Follow-up time**	**n**	**Mean**	**SD**	**Mean**	**SD**^**3**^	**Effect size**	***p*-value**
**NASS**^**4**^								
Pain + disability	< 9 y	63	2.97	0.67	2.67	0.85	0.46	0.004
	≥ 9 y	51	3.13	0.79	2.51	0.82	0.79	< 0.001
Neurogenic symptoms	< 9 y	61	2.40	1.14	2.33	1.02	0.06	0.642
	≥ 9 y	51	2.42	1.05	2.34	1.25	0.08	0.638
**Short-Form 36**								
Physical functioning	< 9 y	59	62.98	17.15	61.18	22.38	-0.11	0.476
	≥ 9 y	40	63.07	16.54	65.81	20.80	0.17	0.418
Physical role	< 9 y	59	47.74	39.11	47.88	43.87	0.00	0.646
	≥ 9 y	40	30.00	35.90	53.13	41.67	0.64	0.005
Bodily pain	< 9 y	59	40.53	14.22	51.92	20.81	0.80	< 0.001
	≥ 9 y	40	36.38	14.97	56.33	19.86	1.33	< 0.001
General health	< 9 y	59	54.34	16.26	53.03	19.84	-0.08	0.541
	≥ 9 y	40	51.43	17.91	52.65	17.08	0.07	0.655
Vitality	< 9 y	59	48.73	15.13	50.17	19.45	0.10	0.508
	≥ 9 y	40	50.38	15.75	52.08	15.24	0.11	0.502
Social functioning	< 9 y	59	75.21	19.76	71.61	23.99	-0.18	0.265
	≥ 9 y	40	69.69	24.82	77.81	18.67	0.33	0.042
Emotional role	< 9 y	59	83.62	30.56	71.19	43.09	-0.41	0.022
	≥ 9 y	40	71.67	39.62	78.33	34.22	0.17	0.376
Mental health	< 9 y	59	67.93	15.96	68.07	17.71	0.01	0.951
	≥ 9 y	40	69.73	18.80	72.65	13.44	0.16	0.270
Physical component summary	< 9 y	59	36.18	8.13	37.97	10.30	0.22	0.166
	≥ 9 y	40	33.95	6.81	38.95	9.69	0.73	0.001
Mental component summary	< 9 y	59	51.01	8.90	48.86	11.51	-0.24	0.086
	≥ 9 y	40	50.28	11.02	51.16	8.93	0.08	0.636

^1^T1: Start of the multidisciplinary biopsychosocial rehabilitation

^2^T3: Long-term follow-up occurring 4–15 years after the multidisciplinary biopsychosocial rehabilitation

^3^SD: Standard deviation

^4^NASS: North American Spine Society Outcome Instrument

### Drop-out analysis

There was no significant difference between patients who were included in the follow-up study and those who were not for age (not included: 64.1 ± 10.1) and gender distribution (not included: 70.3% female).

## Discussion

This prospective study of patients with CLBP after MBR had a substantially longer follow-up period and larger sample size in comparison to previous long-term studies. The results confirmed our hypothesis that patients with CLBP have less pain and disability, as well as a stable quality of life, at a long-term follow-up that was 4 to 15 years following discharge (mean: 9.2 years) compared to before their MBR. The ES for the primary outcome NASS pain + disability remained moderate and was clearly above a minimal clinically important difference [30], and the long-term ES for the SF-36 bodily pain scale was large.

The long-term improvement in pain and disability seen in our study is in line with the majority of the results in four previous studies investigating patients with CLBP [9, 10, 12, 13].

The study of Bendix et al. [9] showed improvements in disability that are in line with our study, but showed no clear, persistent improvement in pain. That study consisted of two subsidiary, randomized controlled trials with five years of follow-up. It included 46 and 37 participants with mean ages of 42 and 41 years. For all participants, continued employment was threatened by their LBP. The MBR program comprised three weeks of five treatment days, followed by three weeks with one treatment day. Pain was measured using a numerical rating scale from 0–10 and disability by a validated PROM for activities of daily living. Only in one subsidiary study did patients’ LBP improve. In both subsidiary studies, patients improved in their activities of daily living. However, there are several important differences with our study, which include a restriction of the age of inclusion to persons younger than 60 years and to patients whose continued employment was threatened by their health condition.

The second study by Cassisi et al. [10] showed improvements in pain and disability similar to our study. That study compared 39 patients with a mean age of 46 years who participated in an intense, 4-week MBR to patients who were referred to an MBR, but did not participate. The median follow-up time was 22.5 months following treatment. Outcome data were collected by interview at the follow-up only. Participants showed significantly higher percentages of pain decreases in a pain rating between 1 and 10 compared to the control group. In addition, the course of functioning that was measured by an interview-based rating system was better in the treatment group. In contrast to our study, the patients retrospectively estimated their pre-treatment status. This may have introduced bias as patients may not reliably remember their previous status. Another possible source of bias was the selection of control groups who declined participation in the MBR, or whose insurance declined coverage. These patients may have also differed from the treatment group in other characteristics that could have affected the outcome.

In the study by Zhuk et al. [13], the MBR had the same duration as our study and also showed improvements in pain and disability. That study followed 59 of 412 eligible patients (14%) with a mean age of 45 years for 10 years after a 3-week MBR. At follow-up, patients showed large improvements on a visual analogue scale for pain compared to the measure at the beginning of the MBR. Moreover, the validated PROM for disability improved significantly. However, in comparison to our study, the follow-up rate was considerably lower. This may have overestimated the effects due to selection bias as patients who were satisfied with the outcome may be more likely to respond.

In line with our study, Patrick et al. [12] have also reported long-term improvements in pain and disability. That study collected data via interviews 13.3 years (SD: 2.1 years) after a 3-week inpatient MBR. Patients had to be not working due to pain for 3 to 30 months. Their mean age was 42 years. Pain and disability were measured by validated PROMS. Data at follow-up were collected by interview and compared to pre-test, post-test and 6-month values. The 26 participating patients from 48 eligible patients had better pain scores and less disability at the long-term follow-up compared to the pre-test, post-test and 6-month scores. In contrast to our study, this study included only patients on sick leave, collected follow-up data by interview, and had a considerably lower sample size.

Overall, the comparison of these four previous studies and ours is limited as all of the studies applied different outcome measures. Furthermore, they differed in terms of patient characteristics, program content, methods of data collection, comparison groups, and the duration of follow-up. Despite these differences with our study, all four studies showed an improvement in disability in line with our study [9, 10, 12, 13]. Three of the four studies confirmed improvements in pain [10, 12, 13], while one study showed inconsistent results in two subsidiary studies [9].

The long-term effects in this study were larger than those in a meta-analysis of randomized controlled trials [5] that reported a mean effect size of 0.21 for pain and 0.23 for disability at a follow-up of at least one year after an MBR. One possible reason for the larger effects in our study are differences in patient characteristics regarding prognostic factors for a beneficial outcome [31], e.g. the low proportion of patients with depressive symptoms, or the omission of an inclusion criterion regarding work disability could have contributed to a better outcome. In addition, the very long follow-up period in this study compared to previous studies could have resulted in larger effects if effects decrease between 3 months and one year but then increase over the long term. This is supported by the long-term study by Patrick et al. [12] that showed a larger effect after 13 years compared to after 6 months. However, the selection of healthier patients who were accessible at the long-term follow-up could have contributed to higher ESs.

The long-term, stable SF-36 scores of the physical function component suggest that an MBR may also improve the long-term course of general health because, in the average population from the age decade 60–69 to the 70–79 decade, these scores decline [32]. These general health benefits may be attributed to a motivation for increased physical activity due to the MBR. However, the long-term results of the SF-36 should be interpreted with caution as two patients were not included in the long-term follow-up due to acute, severe illness and 14 had already died. The exclusion of these patients could have resulted in an overestimation of the favorable long-term course if they had a worse course compared to the included patients.

The differences in the course from T1 to T3 between the NASS pain and disability scale and the SF-36 scales for physical function and physical role are surprising. One reason for these differences is the combination of pain and disability in the NASS scale, as improvements in pain were greater than in disability. This is confirmed by the large improvement in the SF-36 pain scale in this study, as well as by results from a previous study in which the NASS items for pain and disability were analyzed separately [14]. Another reason for the lack of maintained improvement when using these two SF-36 scales could be the increase in the number of comorbidities, which may affect a generic assessment when using the SF-36 as opposed to a disease-specific assessment when using the NASS.

This is the first long-term study after an MBR that measured physiological outcomes. Muscle strength for knee extension and flexion remained stable during the long observational period. Strength training of these muscles is important in patients with LBP because the strength of knee extension, and probably also knee flexion, is reduced in patients with LBP compared to healthy controls [33]. Furthermore, muscle strength of knee extension is associated with persistent lower extremity disability and mortality over the long-term [34].

In addition, mobility as measured by the timed-up-and-go-test and walking ability measured by the 6-min-walk-test were stable. These results suggest that continued physical activity may have counteracted age-related decline in physical performance. Accordingly, continued physical activity could have contributed to the positive, long-term outcome for pain and disability as higher levels of physical activity are associated with less pain [35].

However, it must be considered that the physiological measurements were exploratory, and the subgroup was small. Accordingly, the favorable course of these results could also be due to selection bias or chance. Therefore, these results need confirmation in future studies before firm conclusions can be drawn.

To corroborate the results for the primary outcome of pain and disability, we tested the significance of change with a second statistical method, mixed linear models. This method allows for the modeling of the effects of time, disease severity, age, and sex on the response variables for potentially heterogeneous individuals. The significance of the changes at the long-term follow-up confirmed the favorable long-term course for pain and disability.

It is interesting to note that despite the expected increase in comorbidities during follow-up, pain and disability improved. One possible reason for the maintained improvement is that some comorbidities that become more prevalent with age, such as hypertension or diabetes mellitus, have little impact on condition-specific outcomes. Furthermore, increased physical activity due to the MBR may also reduce potential disability associated with new comorbidities.

### Limitations

An important limitation of this study is the lack of a control group. Accordingly, it is not possible to determine which factors other than the MBR contributed to our long-term results. For example, another MBR during the follow-up could have led to an overestimation of the effects. In contrast, a new onset of osteoporosis and associated pain and disability could have led to an underestimation of the effects. We intended to include as many patients as possible to capture the long-term course in real life. Accordingly, this study showed that after participation in an MBR, there is not necessarily a new deterioration in pain and disability, and improvements can last for many years despite increasing age.

A second limitation is the loss to follow-up of 62% of the participants of the MBR. However, the response rate was in the medium range compared to previous studies with follow-ups lasting many years after an MBR [9–13]. The similar distribution of age and gender in participants and non-participants reduced the risk of bias, yet other factors could have led to a selection bias. For example, moving to a retirement home or the death of patients could be reasons for unsuccessful attempts to contact those with a worse long-term course.

A third limitation is the exclusion of two patients with severe illness at T3. This criterion, and the exclusion of 14 patients who had died, could have led to an overestimation of the benefits as these patients likely had a worse course compared to the included patients. The reason for the exclusion criterion of severe illness was that severe illness at the time of follow-up and the associated disability would have masked the long-term course of pain and disability.

A fourth limitation is the very broad time frame of follow-up between 4 and 15 years. For the primary outcome of pain and disability, the subgroup analysis of patients with less and more than nine years follow-up suggests that this outcome improves significantly independently of the length of follow-up.

The older age of participants in this study compared to other CLBP studies limits the generalizability of the results to younger patients. However, the investigation of CLBP outcomes in older adults is important given that there is still little evidence of effective therapy for CLBP in this population, even though back pain is quite common in this group [36].

Unfortunately, it is an ethical problem to conduct randomized controlled trials to evaluate the long-term course after MBR in CLBP patients because it is unethical withholding patients from a treatment that is recommended in guidelines. This may be an important reason for the lack of recent controlled studies on this topic.

## Conclusion

The results of this study suggest that patients with CLBP benefit from participation in MBR in terms of improved pain and disability for a number of years and perhaps for the remainder of their lives. This is an encouraging finding for patients who consider participating in an MBR (or for those who had participated in an MBR) and are concerned that, following participation, their CLBP may worsen with increasing age. The long-term improvements should be considered by health insurances when making decisions concerning whether to provide coverage for MBR programs.

Future studies with periodic follow-ups could further evaluate those factors that contribute the most to the long-term benefits following MBR, e.g., exercise adherence, sport activities, strategies for stress reduction, ergonomics in daily activity, social factors, co-morbidity, or the social environment.

## Data Availability

The data set is not available because patients did not consent to the use of their data in a public repository.

## Abbrevations

CLBP: Chronic low back pain
ES: Effect size
HADS: Hospital Anxiety and Depression Scale
LBP: Low back pain
MBR: Multidisciplinary biopsychosocial rehabilitation
NASS: North American spine society lumbar spine outcome assessment instrument
PROM: Patient reported outcome measure
SF-36: Short Form 36
